# An Adaptable Two-Color Flow Cytometric Assay to Quantitate the Invasion of Erythrocytes by *Plasmodium falciparum* Parasites

**DOI:** 10.1002/cyto.a.20972

**Published:** 2010-09-24

**Authors:** Michel Theron, Richard L Hesketh, Sathish Subramanian, Julian C Rayner

**Affiliations:** Sanger Malaria Programme, Wellcome Trust Sanger Institute, Wellcome Trust Genome CampusCambridge, United Kingdom

**Keywords:** malaria, *Plasmodium falciparum*, merozoite, host-parasite interactions, flow cytometry, phenotype, erythrocyte invasion

## Abstract

*Plasmodium falciparum* genotyping has recently undergone a revolution, and genome-wide genotype datasets are now being collected for large numbers of parasite isolates. By contrast, phenotyping technologies have lagged behind, with few high throughput phenotyping platforms available. Invasion of human erythrocytes by *Plasmodium falciparum* is a phenotype of particular interest because of its central role in parasite development. Invasion is a variable phenotype influenced by natural genetic variation in both the parasite and host and is governed by multiple overlapping and in some instances redundant parasite–erythrocyte interactions. To facilitate the scale-up of erythrocyte invasion phenotyping, we have developed a novel platform based on two-color flow cytometry that distinguishes parasite invasion from parasite growth. Target cells that had one or more receptors removed using enzymatic treatment were prelabeled with intracellular dyes CFDA-SE or DDAO-SE, incubated with *P. falciparum* parasites, and parasites that had invaded either labeled or unlabeled cells were detected with fluorescent DNA-intercalating dyes Hoechst 33342 or SYBR Green I. Neither cell label interfered with erythrocyte invasion, and the combination of cell and parasite dyes recapitulated known invasion phenotypes for three standard laboratory strains. Three different dye combinations with minimal overlap have been validated, meaning the same assay can be adapted to instruments harboring several different combinations of laser lines. The assay is sensitive, operates in a 96-well format, and can be used to quantitate the impact of natural or experimental genetic variation on erythrocyte invasion efficiency. © 2010 International Society for Advancement of Cytometry

Infection with the malaria parasite *Plasmodium falciparum* causes more than a million deaths each year, largely in children under the age of five. *Plasmodium* lifecycles are complex, involving multiple stages in both vertebrate and invertebrate hosts, but the symptoms and pathology of malaria are all caused by the invasion and multiplication of *Plasmodium* parasites inside vertebrate erythrocytes. The process by which *P. falciparum* recognizes and invades human erythrocytes is therefore the target of extensive study and depends on a number of extracellular receptor–ligand interactions (1). These interactions are overlapping and to some extent redundant, meaning that erythrocyte invasion is a relatively plastic phenomenon that can be influenced by natural genetic variation in both parasite and human genomes. The high frequency of specific erythrocyte receptor variants in some malaria endemic populations, such as the Gerbich (glycophorin C null) phenotype in Melanesian populations, is attributed to an impact on *P. falciparum* invasion efficiencies (2). Similarly, *P. falciparum* isolates collected in the field display a range of invasion phenotypes, presumably due to genetic variability in the expression or sequence of key invasion ligands (3–9).

Despite the abundant evidence for natural genetic variation impacting erythrocyte invasion, there are no well-established examples of natural *P. falciparum* variants being associated with specific invasion pathways. There are two clear road blocks to carrying out large-scale association studies to identify such associations—unbiased genotyping and high throughput phenotyping. *P. falciparum* genotyping approaches are now advancing rapidly and a range of genome-wide tools are being applied and under development (10,11). However, phenotyping platforms have lagged behind these advances, and in the case of erythrocyte invasion has been often dependent on counting *P. falciparum* parasites using microscopy.

Flow cytometry has clear applications to phenotyping malaria parasites, particularly during the intraerythrocytic stages. Because erythrocytes are anuclear, erythrocytes infected with *P. falciparum* can be detected and distinguished from noninfected erythrocytes using DNA dyes, and several cytometric protocols have now been published using flow cytometry to count *P. falciparum* growth using fluorescent DNA dyes (12–14). However, such assays alone cannot be used to phenotype erythrocyte invasion because phenotyping invasion depends not only on counting parasites but also counting which erythrocytes the parasites have invaded. All invasion assays involve adding *P. falciparum* parasites to erythrocytes with a limited subset of erythrocyte receptors (for example, erythrocytes from known human blood group variants or erythrocytes that have been treated with enzymes to remove specific receptors) and scoring parasite density 48 h later. The reason that standard growth assays cannot be applied in this context is because uninfected (and hence untreated) erythrocytes are always present at some level in the starting parasite culture (referred to hereafter as “donor” cells). For invasion to be phenotyped, it is essential that parasites present in donor cells are not counted, whereas parasites that have invaded the erythrocytes with a reduced receptor repertoire (referred to hereafter as “target” cells) are counted.

In previous assays, this fundamental problem in phenotyping invasion has been overcome by one of two approaches. In one, purification methods are used in an attempt to eliminate all uninfected erythrocytes from the donor culture, but purification requires larger volumes of culture and is not suited to high throughput assays of multiple lines. The widely used alternative involves pretreating the donor culture with a combination of enzymes, usually neuraminidase and trypsin, in order to cleave all available erythrocyte invasion receptors. This approach is designed to prevent reinvasion into all uninfected erythrocytes present in the donor culture and limit invasion to the target erythrocytes (15,16). This has recently been successfully combined with a fluorescent DNA dye to allow measurement using flow cytometry (17), but necessarily involves serial manipulation of the *P. falciparum* culture and exposing it to enzymes that are sometimes present in nonphysiological buffers.

To minimize parasite handling, two-color flow cytometry was investigated as an alternative to quantitate erythrocyte invasion. Unlabeled donor *P. falciparum* cultures were coincubated with target erythrocytes that had been labeled with fluorescent dyes, and parasites present in the donor and target population were quantitated using fluorescent DNA dyes. Multiple DNA dyes were tested and protocols adjusted to minimize background. Cell dyes were chosen to have minimal emission overlap with the best performing DNA dyes and to label erythrocytes cytoplasmically rather than on the erythrocyte surface as has been used previously (3), reasoning that surface labels may reduce invasion efficiencies and therefore reduce the sensitivity of the assay. This combinational approach resulted in the identification of several dye combinations that recapitulate known invasion phenotypes, meaning that the assay can be adapted to multiple flow cytometers, depending on the laser lines available. This 96-well plate-based adaptable phenotyping platform should be of broad utility for measuring the impact of natural or experimental genetic variation in either host or parasite on erythrocyte invasion efficiency and could be applied to genotype–phenotype association studies.

## MATERIALS AND METHODS

### In Vitro Culture of *P. falciparum* Parasites

*P. falciparum* parasite strains 3D7, Dd2, and HB3 were routinely cultured in human O+ erythrocytes (NHS Blood and Transplant, Cambridge, UK) at 5% hematocrit in complete medium containing 10% human sera, under an atmosphere of 1% O_2_, 3% CO_2_, and 96% N_2_ (BOC, Guildford, UK). Parasite cultures were synchronized on early stages with 5% d-sorbitol (Sigma-Aldrich, Dorset, UK). Use of erythrocytes from human donors for *P. falciparum* culture was approved by NHS Cambridgeshire 4 Research Ethics Committee.

### Parasite Labeling

Parasite cultures were stained with a DNA dye according to the following protocol. The cells were washed with PBS before staining with 10 μg/mL ethidium bromide (Sigma-Aldrich, Dorset, UK) in PBS, 2 μM Hoechst 33342 (Invitrogen, Paisley, UK) in RPMI 1640 or 1:5,000 SYBR® Green I (Invitrogen, Paisley, UK) in PBS, for 1 h at 37°C. After staining, the cells were washed with PBS, before being fixed with a 2% paraformaldehyde (Sigma-Aldrich, Dorset, UK), 0.2% glutaraldehyde (Sigma-Aldrich, Dorset, UK) solution in PBS for 1 h at 4°C. Finally, the suspension was washed with PBS before acquisition on a flow cytometer. An alternative protocol was used to allow removal of RNA. In this method, the cells were first fixed with a paraformaldehyde/glutaraldehyde solution as described earlier. Following a PBS wash, the cells were permeabilized for 10 min at room temperature with 0.3% Triton® X-100 (Sigma-Aldrich, Dorset, UK) in PBS. The suspension was then washed with PBS before RNase treatment for 1 h at 37°C with 0.5 mg/mL ribonuclease A (MP Biomedicals, Illkirch, France) in PBS. The cells were next washed with PBS before staining with the DNA dyes as described earlier. Finally, the cells were washed with PBS before acquisition on a flow cytometer.

### Erythrocyte Labeling

Erythrocytes were labeled with amine-reactive fluorescent dyes. The required volume of O+ erythrocytes at 2% haematocrit in RPMI 1640 was centrifuged and the pellet resuspended to 2% hematocrit with either 20 μM carboxylfluorescein diacetate succinimidyl ester (CFDA-SE) (Invitrogen, Paisley, UK) or 10 μM 7-hydroxy-9H-(1,3-dichloro-9,9-dimethylacridin-2-one) succinimidyl ester (DDAO-SE) (Invitrogen, Paisley, UK) in RPMI 1640 and incubated for 2 h at 37°C. The suspension was washed with complete medium and the pellet resuspended to 2% hematocrit with complete medium and incubated for 30 min at 37°C. The suspension was then washed twice with incomplete medium (without human sera) and finally resuspended to 2% hematocrit with incomplete medium. The cells were stored until use at 4°C for up to 24 h.

### Flow Cytometry and Data Analysis

Stained samples were examined with a 355 nm 20 mW UV laser, a 488 nm 20 mW blue laser, and a 633 nm 17 mW red laser on a BD LSRII flow cytometer (BD Biosciences, Oxford, UK). Ethidium bromide (EB) was excited by a blue laser and detected by a 610/20 filter. Hoechst 33342 was excited by a UV laser and detected by a 450/50 filter. SYBR Green I and CFDA-SE were excited by a blue laser and detected by a 530/30 filter. DDAO-SE was excited by a red laser and detected by a 660/20 filter. BD FACS Diva (BD Biosciences, Oxford, UK) was used to collect 100,000 events for each sample. FSC and SSC voltages of 423 and 198, respectively, and a threshold of 2,000 on FSC were applied to gate on the erythrocyte population. The data collected was then further analyzed with FlowJo (Tree Star, Ashland, Oregon).

All experiments were carried out in triplicate and the data is presented as the mean ± standard error of the mean. GraphPad Prism (GraphPad Software, La Jolla, CA) was used to plot parasitemia data generated and carry out statistical analysis.

### Microscopy

Standard blood smear microscopy was performed to determine parasitemia. In brief, a small aliquot of culture was smeared on a glass slide, fixed with 100% methanol, and stained with 10% Giemsa solution (Sigma-Aldrich, Dorset, UK). Parasitemia was determined by counting the number of parasitized red blood cells (pRBC) per 1,000 total red blood cells (RBC) examined by oil immersion with a Leica DME microscope (Leica Microsystems, Milton Keynes, UK). All parasitemia represented were the average of three replicates.

Fluorescence microscopy was performed on cells stained with Hoechst 33342, SYBR Green I, CFDA-SE, and/or DDAO-SE, as described above. Cells were examined by oil immersion with either a Leica DM2500 microscope (Leica Microsystems, Milton Keynes, UK) or a Zeiss LSM510 laser scanning system (Carl Zeiss, Welwyn Garden City, UK). Images were captured using either Leica LAS AF (Leica Microsystems, Milton Keynes, UK) with a Leica DFC420C camera (Leica Microsystems, Milton Keynes, UK) or Zeiss LSM Image Browser (Carl Zeiss, Welwyn Garden City, UK).

### Invasion Assay and Enzymatic Treatment of Human Erythrocytes

Invasion assays were carried out in round-bottom 96-well plates, with a culture volume of 100 μL per well at a hematocrit of 2%. Plates were incubated inside an incubator culture chamber (VWR, Lutterworth, UK), gassed with 1% O_2_, 3% CO_2_, and 96% N_2_, and kept at 37°C for 48 h.

Erythrocytes labeled with either CFDA-SE or DDAO-SE as described earlier were pelleted and washed with incomplete media. The pellet was resuspended to 2% hematocrit with incomplete medium and aliquoted into individual microfuge tubes. Neuraminidase from *Vibrio cholerae* (Sigma-Aldrich, Dorset, UK) was added to the appropriate tubes to obtain a final concentration of 20 mU/mL, and all of the tubes were incubated under rotation at 37°C for 1 h. The cell suspensions were pelleted and washed with incomplete media. The pellets were then resuspended to 2% hematocrit with incomplete medium. Trypsin (Sigma-Aldrich, Dorset, UK) or chymotrypsin (Sigma-Aldrich, Dorset, UK) was added to the appropriate tubes to obtain a final concentration of 50 μg/mL (low trypsin) or 1 mg/mL (high trypsin and chymotrypsin), and all of the tubes were incubated under rotation at 37°C for 1 h. The cell suspensions were pelleted and washed twice with incomplete media. The pellets were then resuspended to 2% hematocrit with complete medium, before being added to the culture plate. pRBC were then added to each well and the well suspension mixed before incubation for 48 h. At the end of the incubation period, RBC were harvested and pRBC were stained as described earlier. Data collection and statistical analysis were carried out as described earlier.

Detailed Standard Operating Procedures for all invasion assays are available at http://www.sanger.ac.uk/research/projects/malariaprogramme-rayner/ (Resources section).

## RESULTS

### Detection of pRBC by Flow Cytometry Using Different Fluorescent Dyes

**Figure 1:**
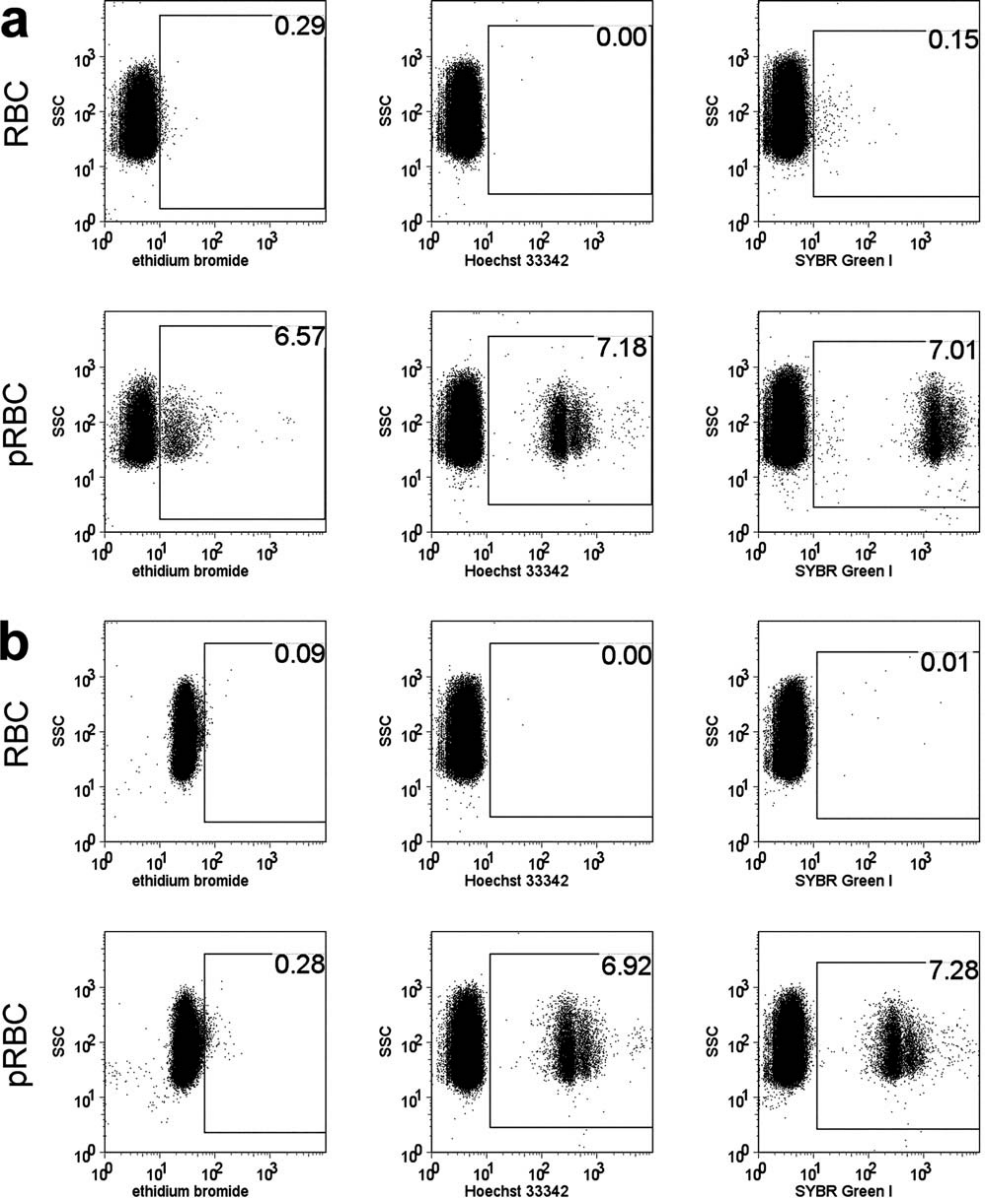
Staining of pRBC with fluorescent DNA-binding dyes. pRBC were detected by flow cytometry after staining with either 10 μg/mL EB, 2 μM Hoechst 33342, or 1:5,000 SYBR Green I. **a**: Uninfected RBC and pRBC were directly stained with the DNA dyes. **b**: Uninfected RBC and pRBC were fixed, permeabilized, and treated with RNase before staining with the DNA dyes.

To develop an assay with the highest sensitivity, several fluorescent dyes were tested under different staining and treatment conditions for their ability to discriminate between parasitized red blood cells (pRBC) and uninfected RBC and to yield accurate and reproducible parasitemia counts. As shown in Figure 1a, staining uninfected RBCs with either SYBR Green I or EB resulted in a low level of background, which could be removed by including an RNase treatment step (Fig. 1b). By contrast, Hoechst 33342 staining yielded minimal background in uninfected RBCs (Fig. 1a). All three dyes identified a population of DNA containing cells (Fig. 1b), which were confirmed as pRBC using fluorescence microscopy (Supporting Information Fig. S1). However, EB staining of pRBC was at a significantly lower intensity than Hoechst 33342 and SYBR Green I under these and all other conditions tested (data not shown), making discrimination between uninfected and infected RBC less robust. Ethidium bromide staining, while used in some assays (3), was therefore not as effective in this case as Hoechst 33342 and SYBR Green I for quantitating pRBC and was excluded from further development.

**Figure 2:**
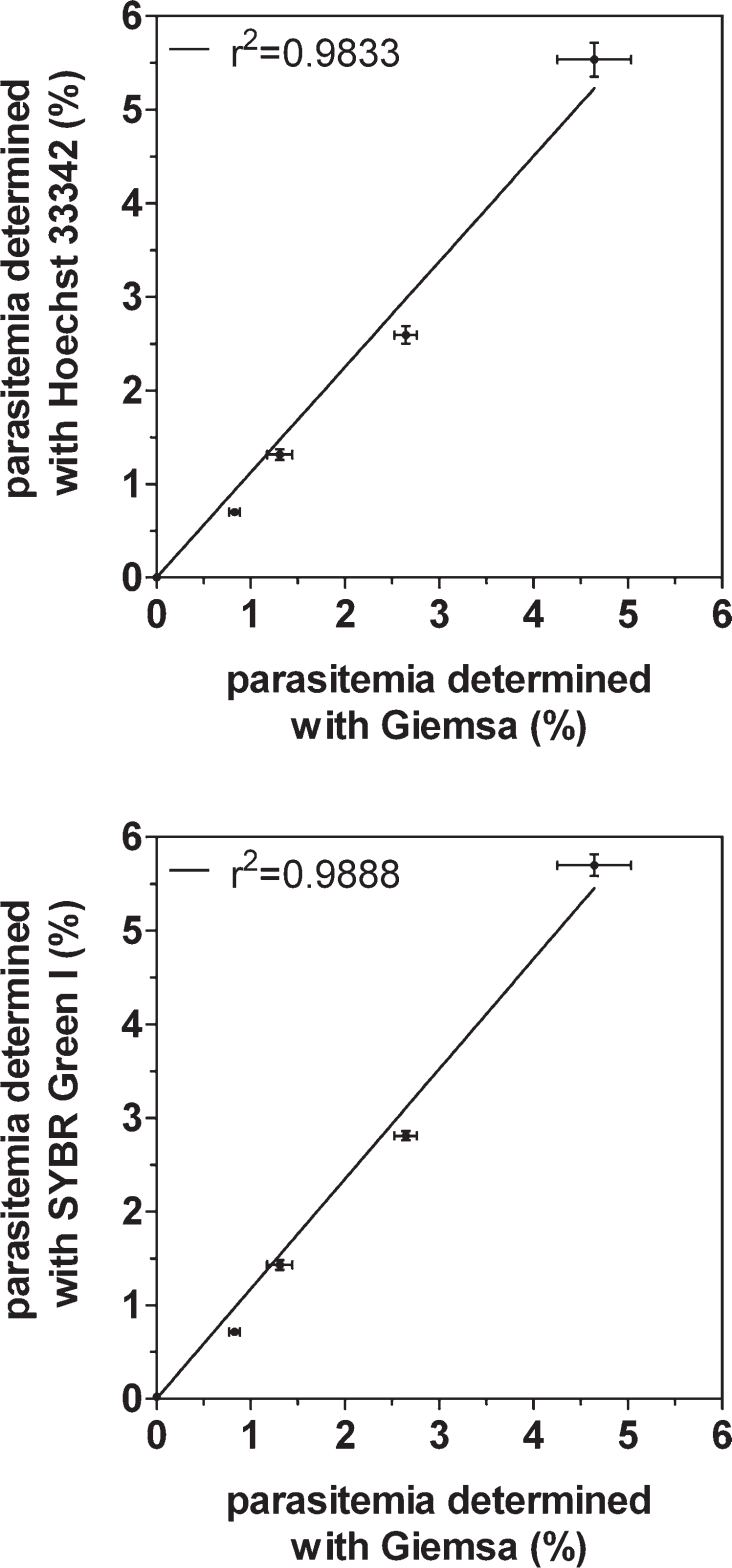
Accuracy of flow cytometry in determining parasitemia. Correlation between parasitemia determined by flow cytometry, using either Hoechst 33342 (direct staining) or SYBR Green I (staining post RNase treatment), and by light microscopy, using Giemsa staining, for a serial dilution of a Dd2 parasite culture. Standard error bars are represented on the horizontal axis for microscopy counts and vertical axis for flow cytometry counts.

To validate the use of either dye in the determination of parasitemia, the accuracy of the flow cytometry counts was compared with counts generated from Giemsa-stained thin smears, the traditional gold standard for parasitemia calculations. A culture of Dd2 strain *P. falciparum* parasites was serially diluted and the resulting parasitemia measured in triplicate by flow cytometry, using either Hoechst 33342 or SYBR Green I, as well as by microscopy. With each fluorescent dye, the flow cytometry-based method provided reproducible counts that correlated well (*r*^2^ = 0.9833 for Hoechst 33342 and *r*^2^ = 0.9888 for SYBR Green I) with those of the microscopy-based method (Fig. 2). Variability between replicates was routinely lower in flow cytometry-based counts (vertical error bars in Fig. 2) than in microscopy-based counts (horizontal error bars in Fig. 2), reflecting the high number of events counted in flow analysis and the elimination of observer-generated errors inherent in microscopy. These and other repeated tests confirm that both Hoechst 33342 and SYBR Green I produce reproducible parasitemia counts by *P. falciparum*, although in the case of SYBR Green I, an RNase treatment step is required to minimize background.

### Labeling of RBC with Fluorescent Dyes

**Figure 3:**
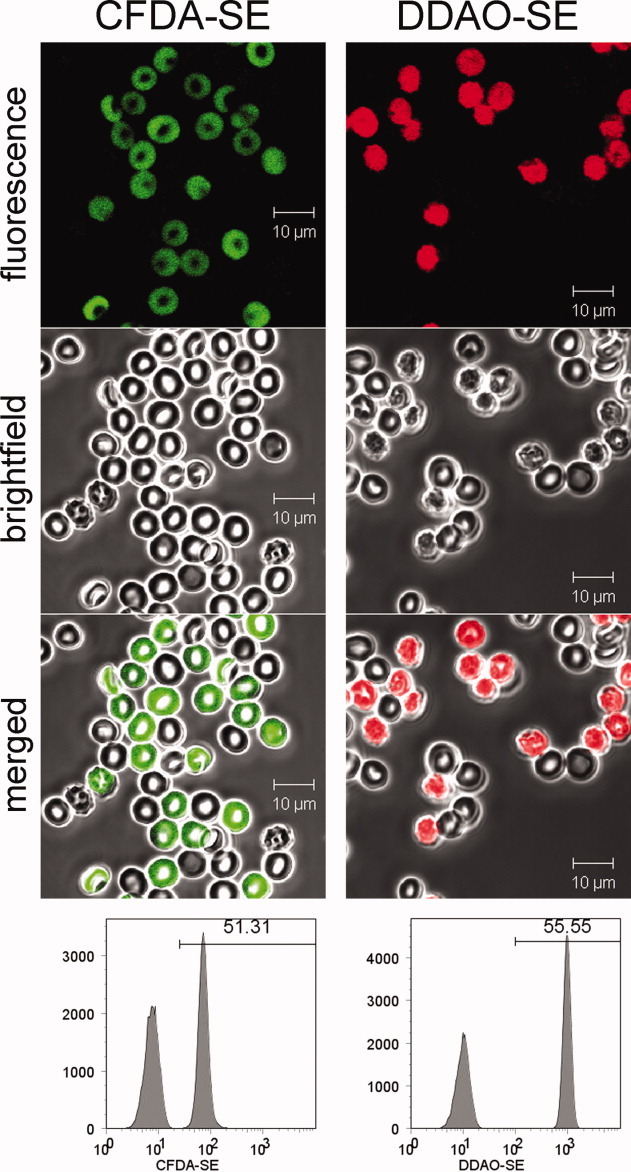
Labeling of target RBC with fluorescent dyes. RBC were labeled with either 20 μM CFDA-SE or 10 μM DDAO-SE and coincubated with an approximately equal quantity of unlabeled RBC for 48 h at +37°C under standard *P. falciparum* culture conditions. RBC were then harvested and fluorescence detected either by confocal microscopy (top panels) or flow cytometry (bottom panels).

As described earlier, phenotyping erythrocyte invasion is distinct from phenotyping *P. falciparum* growth because it requires not only counting RBC that have been invaded but also distinguishing which RBC have been invaded—target RBC (e.g., RBC from specific genetic backgrounds, or that have been enzyme treated) or RBC that are present in the donor *P. falciparum* culture. To investigate the applicability of using fluorescent labels to discriminate between target and donor cells, we tested a wide range of fluorescent cell labels for their ability to label RBC. For the assay to be successful, the fluorescent label must not interfere with parasite invasion or early development, so although membrane labeling dyes such as PKH26 and FITC were tested, particular attention was given to dyes that label cells cytoplasmically and have been shown not to interfere with cell development in other contexts. RBC could readily be labeled with two amine-reactive fluorescent dyes that fit these criteria, CFDA-SE and DDAO-SE. These dyes are membrane permeable but become impermeable after they have entered cells due to cellular esterase activity and a succinimidyl ester group that is able to form covalent attachments to primary amines found in proteins (18). Incubation of RBC with either CFDA-SE or DDAO-SE effectively labeled the whole population, with no detectable unlabelled RBC, a critical consideration for sensitivity (Supporting Information Fig. S2). After coincubation of labeled and unlabeled RBC for 48 h at 37°C, the two populations remained distinct (Fig. 3), indicating that no leakage occurred between labeled and unlabeled RBC. Both dyes are therefore theoretically compatible with a phenotyping assay that involves coincubation of labeled and unlabeled RBC for 48 h during *P. falciparum* cultivation.

### Fluorescent Labeling Has a Minimal Impact on the Ability of *P. falciparum* to Invade RBC

**Figure 4:**
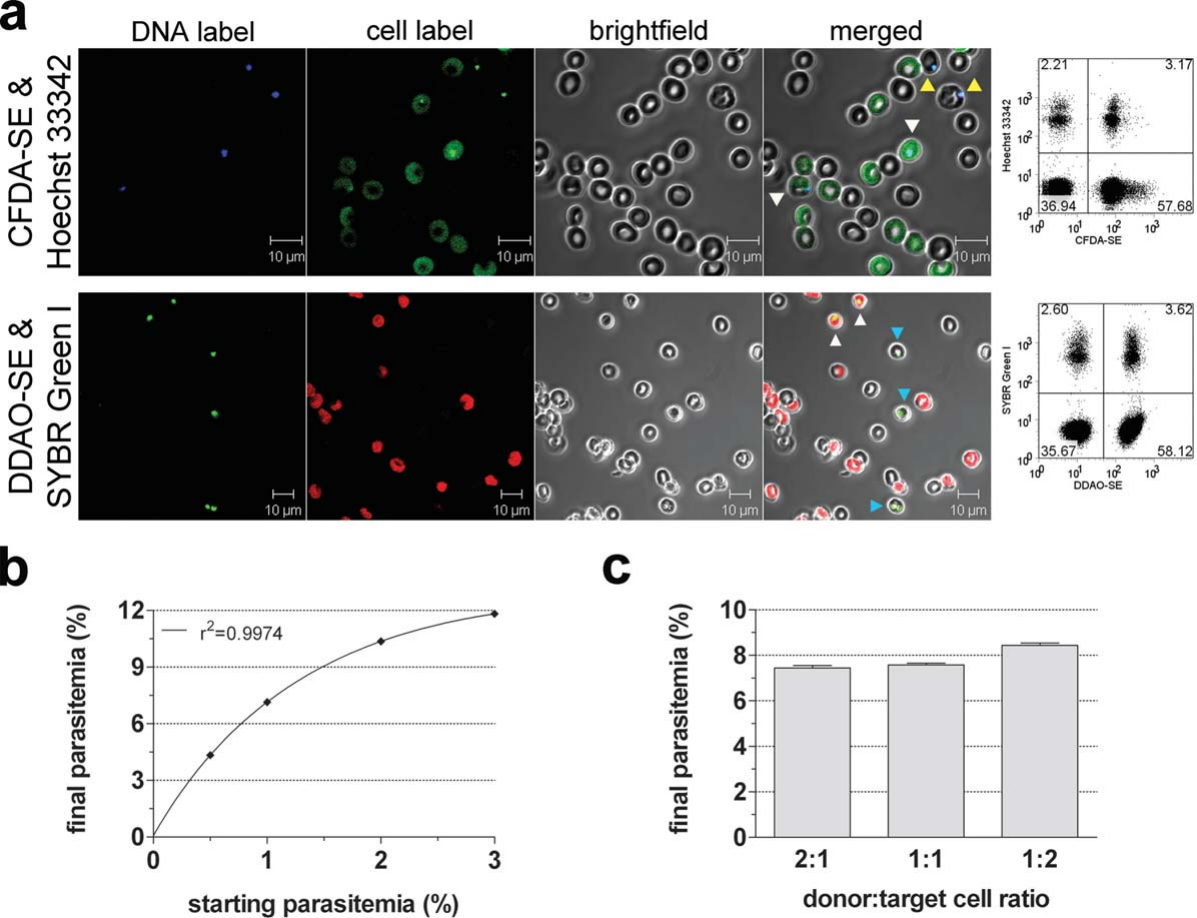
*P. falciparum* invasion in labeled cells. Target RBC labeled with fluorescent dyes CFDA-SE or DDAO-SE were coincubated with unlabeled *P. falciparum* Dd2 strain cultures, containing a mix of uninfected and pRBC, for 48 h under standard *P. falciparum* culture conditions. Cultures were then harvested and stained with Hoechst 33342 or SYBR Green I, respectively. **a**: Cultures were observed by confocal microscopy (first 4 panels) and flow cytometry (last panel on the right). In the microscopy pictures, yellow (Hoechst 33342) and blue (SYBR Green I) arrows point to parasites detected inside unlabeled RBC, whereas white arrows point to parasites detected inside fluorescently labeled RBC. In the dotplot representation of the data generated by flow cytometry, four populations can be readily distinguished: unlabeled, uninfected RBC (lower left); labeled, uninfected RBC (lower right); unlabeled, infected RBC (upper left); and labeled, infected RBCs (upper right). **b**: Effect of the starting parasitemia on the final parasitemia in labeled RBC. Parasitemia of DDAO-SE-labeled RBC was determined by SYBR Green I staining using flow cytometry, for different starting parasitemia using donor unlabeled population with increasing parasitemia. **c**: Effect of the unlabeled to labeled RBC ratio on the final parasitemia in labeled RBC. Parasitemia of DDAO-SE-labeled RBC was determined by SYBR Green I staining using flow cytometry, in a mixed unlabeled donor pRBC/label target RBC culture with a volume of 100 μL and a starting parasitemia of 1%.

To test whether CFDA-SE or DDAO-SE impacted the ability of *P. falciparum* to invade RBC, labeled RBC were incubated for 48 h with unlabeled Dd2-infected pRBC, synchronized at the ring stage of development, to allow all parasites to mature, egress, and reinvade either labeled target RBC or unlabeled RBC present in the donor culture population. Parasites were detected using Hoechst 33342 or SYBR Green I staining, by both fluorescence microscopy and flow cytometry. Labeled RBC that had been invaded by *P. falciparum* parasites were readily identifiable by microscopy, and quantification of invasion into labeled and unlabeled RBC showed no impact of CFDA-SE or DDAO-SE labeling on invasion efficiency (Fig. 4a). By contrast, experiments with other dyes produced a clear inhibitory effect, particularly cell surface labels such as PKH26 and 67 (data not shown), presumably because they reduce RBC recognition and invasion.

Invasion rates were tested at a range of initial parasitemia, and optimum reinvasion was obtained with lower initial parasitemia, with a parasitemia between 0.5 and 1% providing the best compromise in term of reinvasion rate versus final parasitemia (Fig. 4b). By contrast, the availability of labeled RBC had little effect on the final parasitemia, as varying the ratio between labeled and unlabeled cells showed only a minor impact on the final parasitemia (Fig. 4C). This implies that the number of RBC is not limiting in this assay, and that there is no competition effect between labeled and unlabeled RBC, again reinforcing the conclusion that these two fluorescent labels do not affect invasion rates. A starting parasitemia of 0.5–1% and a ratio of 2:1 labeled RBC to unlabeled pRBC was selected as the best conditions to observe and quantitate invasion in target RBC.

### A Two-Color Assay to Phenotype *Plasmodium falciparum* Erythrocyte Invasion

**Figure 5:**
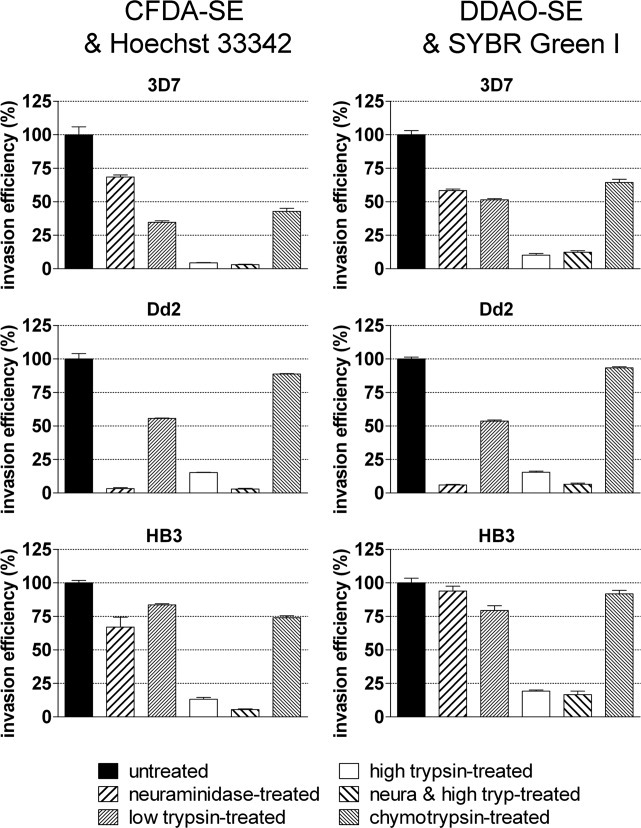
Invasion phenotypes of three laboratory strains of *P. falciparum*. Unlabeled pRBC were incubated with CFDA-SE- or DDAO-SE-labeled RBC, at an unlabeled-to-labeled ratio of 1:2 and a starting parasitemia of 1%. After 48 h, parasites were stained with Hoechst 33342 or SYBR Green I, respectively, and final parasitemia in the target population was determined by gating on the fluorescently labeled RBC population using flow cytometry. Invasion efficiencies were determined as a percentage of the final parasitemia of a mock-treated-labeled positive control RBC group.

To validate this two-color flow cytometric assay, we used it to determine the invasion phenotypes of three laboratory strains of *P. falciparum*, 3D7, Dd2, and HB3, which have all had invasion profiles generated previously by multiple laboratories. CFDA-SE or DDAO-SE labeled RBC were treated with neuraminidase, trypsin, or chymotrypsin in a standard manner and coincubated for 48 h with pRBC. Parasitemia in labeled RBC was then quantitated using Hoechst 33342 (in the case of CFDA-SE labeled RBC) or SYBR Green I (in the case of DDAO-SE labeled RBC). Invasion efficiency was calculated by dividing the parasitemia observed in labeled RBC of a given treatment group by the parasitemia observed in labeled RBC of the mock-treated positive control. For all three strains, the effects of enzyme treatment on invasion efficiency were identical using either CFDA-SE- and DDAO-SE-labeled RBC. For example, Dd2 parasites are unable to invade neuraminidase-treated RBC but are not affected by chymotrypsin treatment (Fig. 5). The phenotypes for each strain were in keeping with the phenotypes determined previously using slide microscopy-based invasion assays: 3D7 is known to be neuraminidase-resistant, trypsin- and chymotrypsin-sensitive, Dd2 chymotrypsin-resistant, neuraminidase- and trypsin-sensitive, and HB3 neuraminidase- and chymotrypsin-resistant, trypsin-sensitive (19). Two-color flow cytometry-based phenotyping of *P. falciparum* erythrocyte invasion therefore recapitulates established invasion profiles.

## DISCUSSION

Flow cytometry is being increasingly used for the quantitation and phenotyping of *P. falciparum* parasites, ranging from parasite growth (12–14) to more complex phenotypes such as oxidative stress (20). In this manuscript, an adaptable platform for phenotyping *P. falciparum* erythrocyte invasion using two-color flow cytometry is described. A primary aim of the development process was to create a platform that could be of the broadest utility in malaria research and therefore across the widest range of instrumentation.

Using flow cytometry to quantitate parasite growth has wide-ranging uses for high-throughput applications such as drug discovery (21,22). Multiple fluorescent DNA dyes have now been used to quantitate parasite growth, including EB (3,23,24), Hoechst 33342 (12,24), SYBR Green I (13,17,21,22), and SYTOX Green (14). In the course of testing dyes for the development of this invasion phenotyping platform, these as well as other options were considered (Vybrant DyeCycle Green, Vybrant DyeCycle Ruby, DRAQ5, LDS751, SYBR Safe, data not shown), but Hoechst 33342 and SYBR Green I yielded the strongest fluorescence and clearest separation of infected and uninfected red blood cells. Of the two compounds, Hoechst 33342 has the significant advantage of producing minimal background. By contrast, the advantage of SYBR Green I is its stimulation by a 488 nm laser that is more standard across all ranges of instrumentation, including less expensive benchtop models that use solid state lasers. However, with SYBR Green I staining, an additional step of RNase treatment is necessary to completely eliminate background in uninfected RBC, presumably due to low-level binding of SYBR Green I to ribosomal and mRNA present in mature RBC (25). This background certainly does not prevent the use of SYBR Green I for quantitation without RNase treatment (17), but omitting this step may have an effect on sensitivity at low parasitemia.

While using fluorescent DNA dyes for quantitation of *P. falciparum* growth is now common, scoring growth is not sufficient to phenotype erythrocyte invasion, which requires a distinction between parasites that have invaded target RBC of interest from those that have invaded donor RBC present in the starting culture. The assay presented here uses fluorescent labels and two-color flow cytometry to distinguish invasion into target and donor RBC. By contrast, the current standard approach to phenotyping invasion is not to label target RBC but instead to pretreat the donor *P. falciparum* cultures with a combination of enzymes designed to prevent all invasion into uninfected donor RBC (15). This approach is certainly effective and is in wide use (4,15,16) but inevitably involves multiple manipulations of the starting culture. Minimizing parasite handling is clearly a desirable feature of any phenotyping approach and has particular relevance when phenotyping recently adapted or even nonadapted field isolates, which are not as robust as commonly used lab strains. In addition to sample handling issues, pretreatment of the starting culture exposes the *P. falciparum* culture to enzymes present in nonphysiological buffers, such as neuraminidase which is often present at low pH. Exposing *P. falciparum* cultures to neuraminidase at concentrations widely used in pretreatment protocols can have an impact on parasitemia, which may impact the dynamic range of pretreatment-based assays (Supporting Information Fig. S3). Finally, multiple enzyme treatment of the donor culture is not always 100% effective in preventing invasion, and low levels of invasion can be observed even in double treated cells in invasion assays, which may decrease sensitivity in an assay depending on pretreatment of donor cultures (15,26).

The two-color flow cytometry approach described here avoids many of these concerns. It requires no manipulation of the starting *P. falciparum* culture other than dilution to the desired parasitemia, it avoids exposure of the culture to anything other than *P. falciparum* growth medium, and it maximizes sensitivity by counting only invasion events into target enzyme-treated RBC. A similar approach has been used previously (3) combining FITC labeling with EB staining. However, FITC predominantly labels the cell surface, which in our experience has a slight inhibitory effect on invasion, again reducing assay sensitivity (data not shown). CFDA-SE and DDAO-SE have the advantages of labeling cells cytoplasmically, and competition experiments showed that these have no effect on parasite invasion. By trialing a number of fluorescent dyes, two DNA dyes and two cell labels were identified as functional for the assay, allowing a combinational approach to phenotyping depending on the characteristics of the available machinery. This assay has clear applications to measure the quantitative impact of experimental or natural genetic variation in host or parasite on erythrocyte invasion, and could be used in genotype–phenotype association studies. It could also be applied to high throughput screening approaches for invasion-inhibitory compounds, although care would need to be taken regarding any fluorescent properties of inhibitors that could interfere with the sensitivity of the assay.
