# Supplementary figures and images for: An Adaptable Two-Color Flow Cytometric Assay to Quantitate the Invasion of Erythrocytes by Plasmodium falciparum Parasites
Source: Cytometry A. 2010 Sep 24;77A(11):1067–74. doi: 10.1002/cyto.a.20972 (PMC3047707; doi:10.1002/cyto.a.20972)

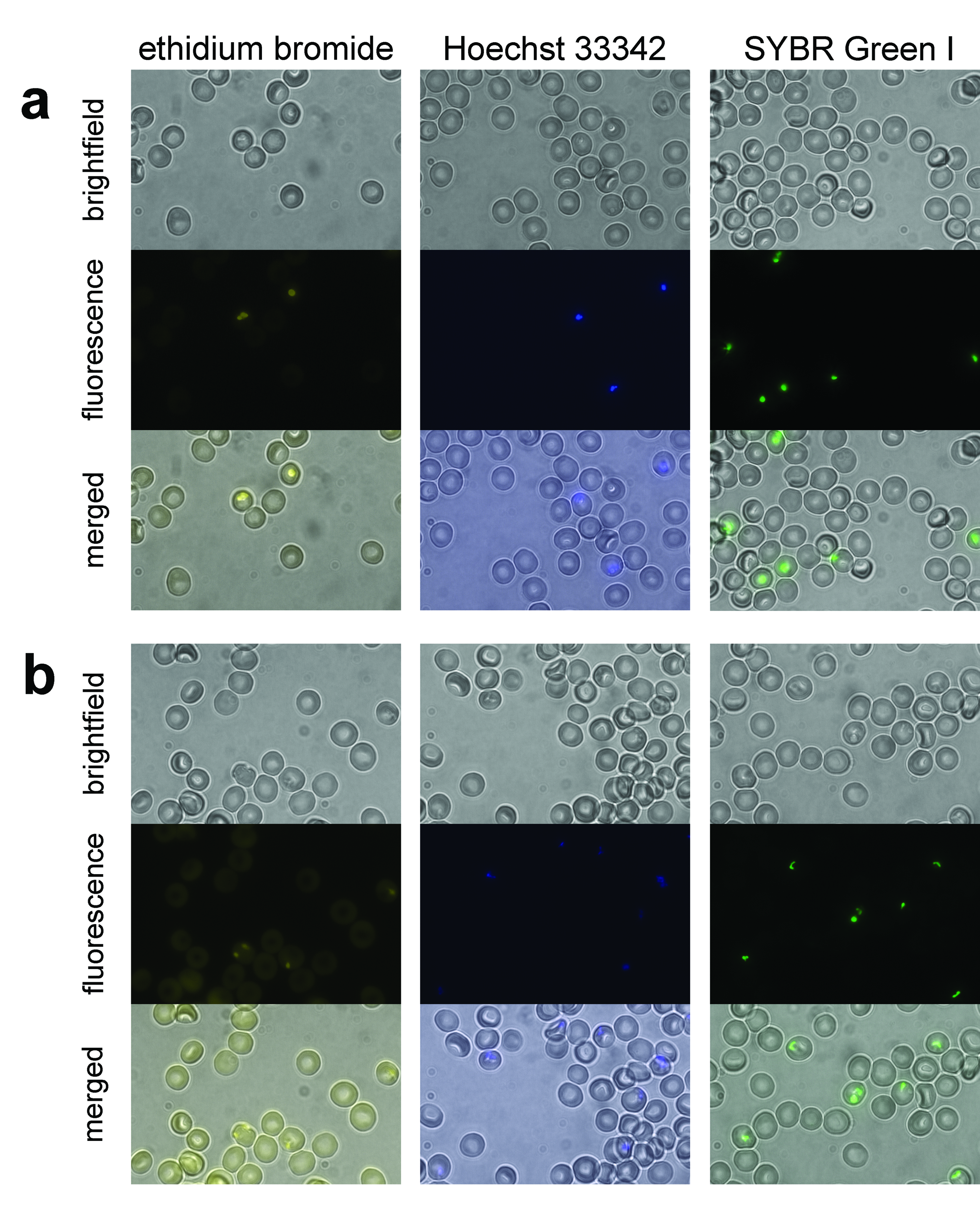

Supplement: Supplementary file 1 [file cyto077A-1067-SD1.tif]

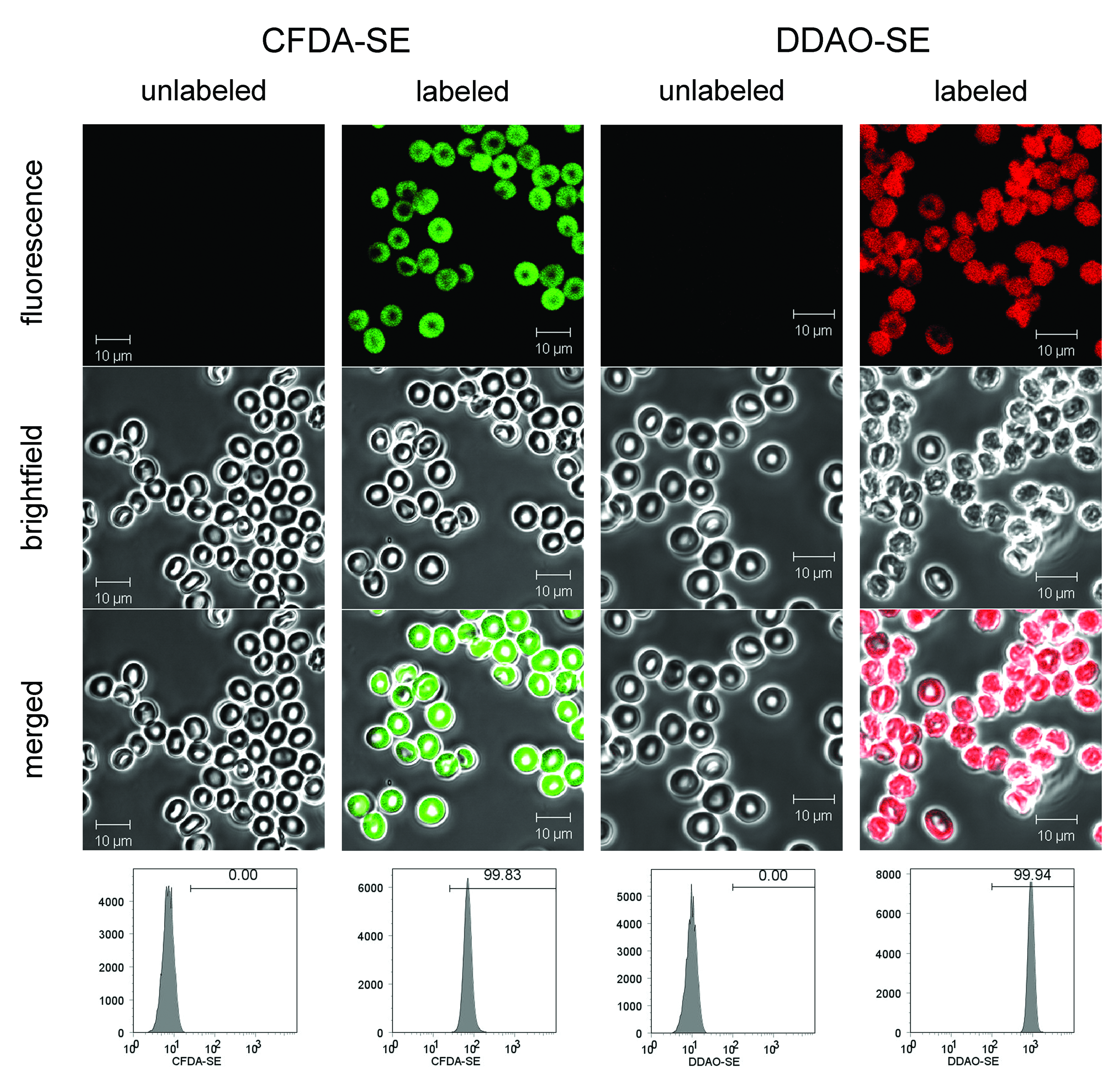

Supplement: Supplementary file 2 [file cyto077A-1067-SD2.tif]

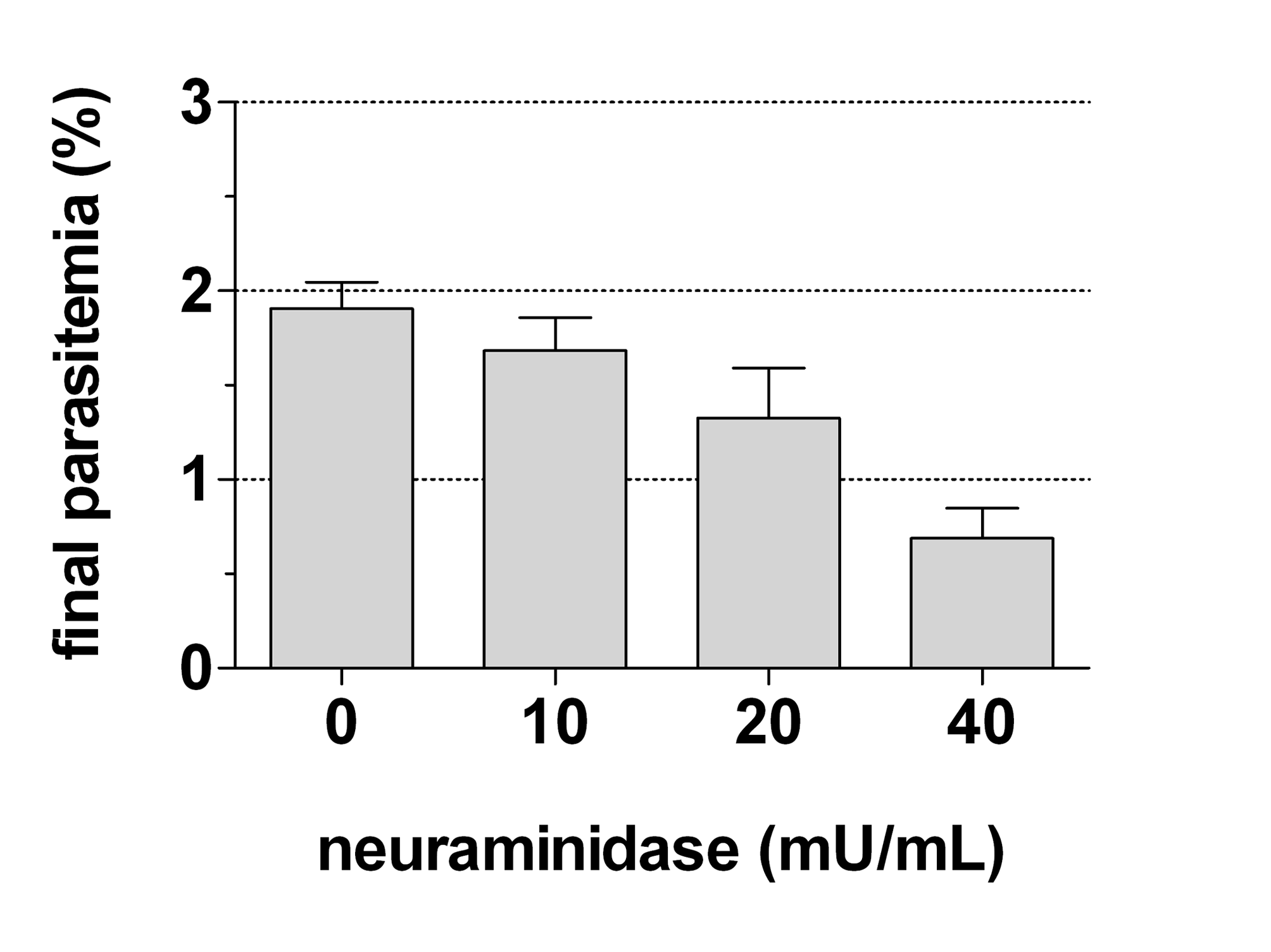

Supplement: Supplementary file 3 [file cyto077A-1067-SD3.tif]
